# Dichotomous branching: the plant form and integrity upon the apical meristem bifurcation

**DOI:** 10.3389/fpls.2014.00263

**Published:** 2014-06-06

**Authors:** Edyta M. Gola

**Affiliations:** Department of Plant Developmental Biology, Institute of Experimental Biology, University of WrocławWrocław, Poland

**Keywords:** dichotomy, terminal branching, apical cell, apical meristems, meristem bifurcation

## Abstract

The division of the apical meristem into two independently functioning axes is defined as dichotomous branching. This type of branching typically occurs in non-vascular and non-seed vascular plants, whereas in seed plants it presents a primary growth form only in several taxa. Dichotomy is a complex process, which requires a re-organization of the meristem structure and causes changes in the apex geometry and activity. However, the mechanisms governing the repetitive apex divisions are hardly known. Here, an overview of dichotomous branching is presented, occurring in structurally different apices of phylogenetically distant plants, and in various organs (e.g., shoots, roots, rhizophores). Additionally, morphogenetic effects of dichotomy are reviewed, including its impact on organogenesis and mechanical constraints. At the end, the hormonal and genetic regulation of the dichotomous branching is discussed.

## INTRODUCTION

Regular branching allows plants to expand and adapt to the environment. There are two major types of shoot branching: lateral (axillary), which involves the formation of a primordial bud in the organogenic zone of the apex, and terminal (dichotomous), which is an outcome of the meristem bifurcation. Root branching is mostly related to the initiation of lateral roots in the pericycle or endodermis and only in some plant groups it is a result of a dichotomous division (Evert, 2006). The most common and also the best-studied are axillary shoot branching and lateral root formation, and not much attention is paid to dichotomy, which requires the drastic reorganization of the entire meristem, while not disrupting its integrity upon the division, and as such it has to be tightly controlled. Thus, revealing the mechanisms employed to protect the meristem integrity and function, especially in the actively dividing apices, is extremely interesting. So far the research was mostly focused on the anatomy of dichotomizing apices but the regulation of this underestimated phenomenon requires the elucidation.

## DEFINITION OF DICHOTOMY

Dichotomy means division into two parts and mostly refers to the bifurcation of thalli and axial organs (shoots, roots), giving rise to two morphologically similar yet autonomous parts. Although dichotomy seems to be intuitively easy to define in terms of external morphology, its development and the proper classification are not always clear. In addition, it is present in various plant groups, which differ in the internal organization of growing points (meristems), where the branching is initiated. Accordingly, the definitions of dichotomy and mechanisms involved in the meristem division reflect these structural varieties.

Generally, the apical meristem can consist of one morphologically distinct apical cell (AC), localized at the summit of the meristem, which divisional activity produces all cells and tissues (Evert, 2006). The dichotomy here is defined in a narrow sense as an equal longitudinal division of this single AC, where both derivative cells become the initials for twin apices (Goebel, 1928; Troll, 1937; Schoute, 1938; Bierhorst, 1977). Alternatively, the meristem comprises of one or more groups of morphologically similar initial cells and their youngest derivatives (Evert, 2006). The dichotomy is understood here in a broad sense, as an equal division of the initial zone of such meristems, including initial cells and organizing center (Steeves and Sussex, 1989). This definition of dichotomy is however, often applied also to the meristems with a single AC (Hagemann and Schulz, 1978; Hagemann, 1980).

## MECHANISM OF DICHOTOMOUS BRANCHING

Structural analyses of dichotomizing apices showed that dichotomy can proceed according to different developmental patterns. In the meristems with a single AC it can be a: (1) direct division of the AC, (2) formation of a new branch near the original AC, which remains active in the second branch, and (3) inactivation of the original AC with the simultaneous initiation of the new branch initials. In plants with two ACs, dichotomy is related to repeated divisions of initials (4). In meristems containing one or more groups of initial cells, the entire meristem divides to form dichotomous apices (5).

### DIRECT DIVISION OF AN AC (FIGURE 1A, TABLE 1)

**FIGURE 1:**
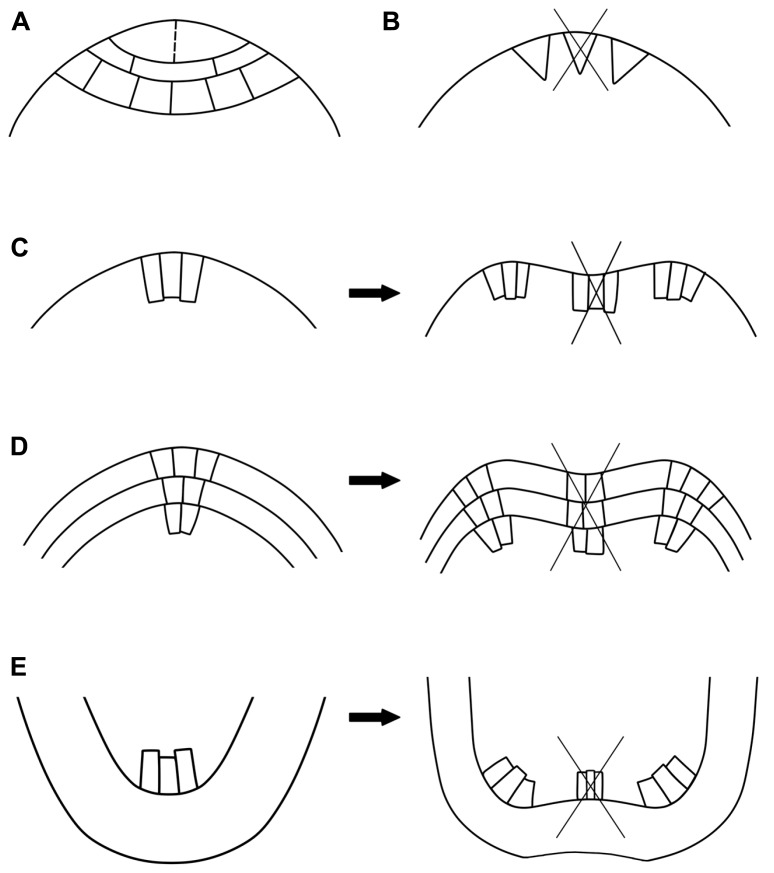
**Mechanisms of dichotomous branching. (A)** *Dictyota dichotoma* type: an equal longitudinal division (marked with a dashed line) of the single apical cell. **(B)** Inactivation of the original apical cell (crossed triangle) followed by the simultaneous initiation of the branch initials (two triangles next to the original apical cell). **(C–E)** Dichotomy in the meristems with one **(C)** or more **(D,E)** groups of initial cells. The meristem zonation is maintained during dichotomy, but the number of cells increases due to intensive cell proliferation. Centrally located cells of the original meristem start to differentiate (crossed groups of cells), separating the dichotomous apices at the flanks of the original meristem. **C,D** – shoot apices, **E** – roots.

**Table 1:** A list of plant species characterized by dichotomous branching.

Plants	Reference and comments
**1. Direct division of an apical cell**	
*Dictyota dichotoma* (Phaeophyta)	Oltmanns (1889, 1904), Goebel (1928), van den Hoek et al. (1995)
*Dennstaedtia* and *Microlepia* (ferns)	White and Turner (1995)
**2. Original AC maintained, becomes an initial for a branch – pseudodichotomous branching**	
*Psilotum nudum*	Roth (1963): aerial stems; interpreted as dichotomy
Metzgeriales, Jungermanniales (liverworts)	Schuster (1984a): pseudodichotomy
**3. Inactivation of the original AC followed by the initiation of ACs for dichotomized apices**	
Gleicheniaceae	Hagemann and Schulz (1978)
Leptosporangiate ferns	Hébant-Mauri (1993)
*Pteridium aquilinum*	Dasanayake (1960), Gottlieb and Steeves (1961)
*Lygodium*	Mueller (1982)
*Osmunda*	Steeves and Sussex (1989)
*Psilotum nudum*	Takiguchi et al. (1997): aerial shoots
*Selaginella kraussiana, S. wallacei, S. martensii*	Webster and Steeves (1964, 1967): rhizophores interpreted as roots
*S. wildenovii*	Cusick (1953)
*S. martensii*	Jernstedt et al. (1994), Lu and Jernstedt (1996)
*S. uncinata, S. delicata, S. caudata, S. plana*	Imaichi and Kato (1989, 1991), Kato and Imaichi (1997), Imaichi (2008): shoots and rhizophores
*S. kraussiana*	Otrcęba and Gola (2011)
*Isoëtes*	Yi and Kato (2001): roots
**4. Intensive divisions of one or two ACs**	
*Selaginella kraussiana*	Harrison et al. (2007)
Anthoceros	Schuster (1984b): formation of the dichotomously lobed thalli due to divisions of one or more prismatic initials in the notch meristem
*Fucus* (Phaeophyta)	Moss (1967): AC in the notch produces few derivatives, which form the forkation; interpreted as pseudodichotomy; van den Hoek et al. (1995): pseudodichotomy
**5. Dichotomy in meristems with multiple initial cells**	
Lycopodiaceae s.l., *Lycopodium*, *Huperzia lucidula*	Troll (1937, 1948), Härtel (1937), Schoute (1938), Imaichi (2008), von Guttenberg (1966), Øllgaard (1979, 1990), Gola and Jernstedt (2011)
*Pinus radiata*	Riding (1976): shoots, ca. 1% of seedlings
*Abies balsamea*	Zagórska-Marek (1985): seedlings
*Cycadaceae, Macrozamia*	Ahern and Staff (1994): ectomycorrhizal roots
*Pinus sylvestris, P. resinosa, P. strobus, P. pineaster, P. radiata*	Ectomycorrhizal roots: Robertson (1954), Wilcox (1968), Faye et al. (1981), Wilson and Field (1984), Piche et al. (1982), Kaska et al. (1999)
*Hyphaene*	Hallé et al. (1978), Tomlinson (1979)
*Nypa fruticans*	Tomlinson (1971)
*Chamaedorea cataractarum*	Fisher (1974)
*Eugeissona*	Fisher et al. (1989): supposedly dichotomy
*Flagellaria indica*	Tomlinson (1970), Tomlinson and Posluszny (1977)
*Strelitzia reginae*	Fisher (1976)
*Asclepias syriaca*	Nolan (1969)
*Mammillaria*	Craig (1945), Boke (1976)
*Echinocereus reichenbachii*	Boke and Ross (1978)
*Edgeworthia chrysantha*	Iwamoto et al. (2005): trichotomy

The classical example of dichotomy in a narrow sense, caused by an even longitudinal division of the AC, is a thallus bifurcation in an alga *Dictyota dichotoma* (Oltmanns, 1889; Goebel, 1928; van den Hoek et al., 1995). However, in most plant species with a single AC, this tetrahedral, lens- or wedge-shaped AC rarely undergoes a perfect longitudinal division, probably due to the reduction of the energy costs related to the new cell wall insertion during cell division (Schüepp, 1966; Cooke and Paolillo, 1980; Barlow, 1992). As a consequence, direct division of the AC is uncommon. It was reported to occur only in some ferns (Bierhorst, 1977). But even this interpretation was later criticized, mostly because the ACs for the new branches originated by formative divisions and not by a direct dissection of the AC (see Lyndon, 1998 for discussion).

### ORIGINAL AC BECOMES AN INITIAL FOR A BRANCH (TABLE 1)

In this pattern, the initial for a new branch originates not by the split of the original AC, but due to formative division in the adjacent segment. At the same time, the original AC maintains the growth of the main axis. Thus, it should be considered the pseudodichotomy (Schuster, 1984a), meaning that even if it looks like dichotomy, it is not formed by the meristem division. Such a branch development was described in detail in thalloid liverworts (Schuster, 1984a); in *Psilotum nudum* it was interpreted as dichotomy (Roth, 1963).

### INACTIVATION OF THE ORIGINAL AC FOLLOWED BY THE INITIATION OF BRANCH INITIALS (FIGURE 1B, TABLE 1)

Dichotomy in a broad sense is applied here, understood as the division of the whole initial zone. The AC, which is usually distinct in non-dividing shoots, ceases its divisional activity prior to dichotomy and becomes indistinguishable at the apex. Then, two new initials are simultaneously initiated next to the inactivated original AC (Hagemann and Schulz, 1978; Hagemann, 1980; Mueller, 1982; Steeves and Sussex, 1989; Imaichi and Kato, 1991; Jernstedt et al., 1994; White and Turner, 1995; Lu and Jernstedt, 1996; Kato and Imaichi, 1997; Imaichi, 2008). This type of dichotomy occurs in shoots, rhizomes and roots, in some ferns and lycophytes (**Table 1**). In details, the changes of the meristem structure were analyzed in the rhizophores - the unique axial organs of *Selaginella*, bearing the root primordia at the tip (Imaichi and Kato, 1989, 1991; Jernstedt et al., 1994; Lu and Jernstedt, 1996; Kato and Imaichi, 1997). Such a dichotomy occurs also in the aerial shoots of *Psilotum nudum*, whereas in irregularly branched subterranean rhizomes, numerous ACs present at the apex can at random be selected for new branches or be inactivated. The mechanism differentiating the fate of ACs in the shoot and rhizome is not known (Takiguchi et al., 1997).

### INTENSIVE DIVISIONS OF TWO ACS FOLLOWED BY THE SELECTION OF BRANCH INITIALS (TABLE 1)

In shoots of *Selaginella kraussiana* two transient ACs are responsible for the apex growth. Here, before dichotomous branching the initial cells divide several times, producing a group of meristematic cells. Then, new ACs are selected for the two resulting axes (Harrison et al., 2007). Possibly, a similar branching pattern is also present in the notch meristems of *Anthoceros*, where intensive divisions of prismatic initial(s) result in a group of meristematic cells, which next split to form the furcated lobes (Schuster, 1984b).

### DICHOTOMY IN MERISTEMS WITH MULTIPLE INITIAL CELLS – SPLIT OF THE ENTIRE MERISTEM (FIGURES 1C–E, TABLE 1)

This mechanism is typical of Lycopodiaceae s.l., where the entire meristem can divide into two even (isotomy) or uneven (anisotomy) parts (**Figure 1C**; Troll, 1937, 1948; Schoute, 1938; Øllgaard, 1979, 1990). In seed plants, dichotomy rarely occurs in gymnosperm shoots (Riding, 1976; Zagórska-Marek, 1985). However, the potential to branch dichotomously is preserved in conifers, as after colonization by mycorrhizal fungi, the lateral roots start to bifurcate (**Figure 1E**; Robertson, 1954; Wilcox, 1968; Faye et al., 1981; Piche et al., 1982). In angiosperms, shoot dichotomy is reported as a typical branching pattern only in several species, mostly in monocotyledons (**Figure 1D**).

Regardless of the structural differences related to the type of organ and organization of its meristem, the morphogenetic processes that accompany the branching are similar in all these plant groups. Dichotomy affects the apex geometry, forces the reorganization of the meristem structure, changes its divisional activity and cell differentiation, and has an impact on organogenesis (e.g., leaf initiation). Its first symptom is broadening of the apex in the plane perpendicular to the future division. In this early stage, the meristem zonation pattern is not disrupted, but the number of cells and volumes of particular meristematic zones increase by intensive cell proliferation. The distinctiveness of superficial layers is maintained during dichotomy progression, as well as the continuity of procambium and vascular tissues in the parental and dichotomous axes. Differentiation of the meristematic cells located between dichotomizing apices ceases the growth of the central part of the original meristem starting the separation of both branches (Härtel, 1937; Nolan, 1969; Tomlinson, 1970, 1971; Boke, 1976; Tomlinson and Posluszny, 1977; Faye et al., 1981; Piche et al., 1982; Laajanen et al., 2007; Raudaskoski and Salo, 2008; Gola and Jernstedt, 2011).

Broadening of the shoot apex can affect the organogenesis due to increasing size of the region of organ initiation. If the leaf initiation precedes the apex dichotomy, the leaf can encircle the enlarged stem (as in *Flagellaria* and* Strelitzia*; Tomlinson, 1970; Fisher, 1976) or be inserted in the increased meristem circumference. In the latter case, the pattern of leaf arrangement (phyllotaxis) can change (Schoute, 1938; Zagórska-Marek, 1985; Gola, 1996). With dichotomy progression, the initiation of new organs is usually maintained at the shoot circumference, but at the inner surface of furcation, organogenesis is repressed until the division is completed. It can be presumed, based on the analogy to the concave apices (Lintilhac, 2014), that this inner surface remains under the tension, which prevents the bulging of primordia. The restoration of organ initiation becomes possible after changes of the physical constraints and the local surface relaxation, similarly to the proposed buckling mechanism of leaf primordia formation (Green, 1996).

## REGULATORY MECHANISMS FOR DICHOTOMOUS BRANCHING

Dichotomous branching is a complex process which requires a precise control of morphogenetic events to maintain the meristem integrity during division. The lack of such a control can lead to unbalanced cell proliferation and, e.g., result in fasciation, to which dichotomy was sometimes compared (Schoute, 1936; Gorter, 1965). Fasciation is usually characterized by flattened stems with multiplied lateral organs; the stems can split to numerous normal or malformed shoots. It is an unpredictable process, caused by various agents (e.g., mutations, chemicals, pathogens including *Rhodococcus fascians*), which is related to the impairment of the hormonal balance and cell proliferation at the meristem (e.g., Gorter, 1965; Leyser and Furner, 1992; Fambrini et al., 2006; Stes et al., 2013). Conversely, dichotomy is a repetitive process of strictly controlled divisions of the entire meristem. Not much is however, known about the regulatory mechanisms at the hormonal and genetic levels, mostly because dichotomy occurs in plants which are not model organisms. Relatively more information is available on the hormonal regulation of the root dichotomy in gymnosperms, due to the intensive research on mycorrhiza.

### HORMONAL CONTROL OF DICHOTOMOUS BRANCHING

The ability to form dichotomous roots seems to be an inherent feature in Pinaceae (Robertson, 1954; Wilson and Field, 1984; Kaska et al., 1999). The intensification of the process, with repeated dichotomies resulting in so-called coralloid structures, is related to the colonization of roots by ectomycorrhizal fungi (Peterson and Bonfante, 1994). The fungal symbionts are probably the source of plant growth regulators, which stimulate morphogenetic changes, including root swelling and dichotomous branching (Barker and Tagu, 2000; Brundrett, 2002). Similar changes in the root architecture can be induced in the absence of fungi by exogenously supplied phytohormones or their inhibitors (Wilson and Field, 1984; Rupp et al., 1989; Kaska et al., 1999; Laajanen et al., 2007; Raudaskoski and Salo, 2008). Application of auxin transport inhibitors [*N*-(1-naphtyl)phthalamic acid (NPA); 2,3,5-triiodobenzoic acid (TIBA)], ethylene precursor [(1-aminocyclopropane-1-carboxylic acid (ACC)], or ethylene releasing compounds [2-chloroethylphosphonic acid (CEPA)] stimulates extensive dichotomous branching of *Pinus* roots, increasing the percentage of coralloid structures up to 25–30% (Kaska et al., 1999). It is suggested that the balance between auxin [indole-3-acetic acid (IAA)] and cytokinin, possibly mediated by the ethylene level, has a regulatory role in this process (Rupp et al., 1989; Kaska et al., 1999; Barker and Tagu, 2000; Laajanen et al., 2007; Raudaskoski and Salo, 2008). Possibly, the NPA-treatment increases the auxin concentration at the root tip, whereas the moderate level of the hormone at the meristem flanks induces dichotomous root formation. At the same time, high IAA concentration at the root tip stimulates the biosynthesis of ethylene, leading to cell differentiation in the central part of the original meristem and consequently, separation of dichotomous roots (Laajanen et al., 2007; Raudaskoski and Salo, 2008).

This mechanism of hormonal regulation corresponds well with the morphogenetic changes in dichotomizing roots of *Pinus*, but because it was proposed based on the auxin distribution in a model plant *Arabidopsis*, it should be validated. Nevertheless, it seems likely that specific hormone distribution and/or concentration can be a universal aspect of dichotomy regulation, as e.g., auxin shapes different developmental processes in all vascular plants and bryophytes (Cooke et al., 2002). In addition, it has recently been shown that the ratio between IAA and cytokinin regulates the dichotomous root branching in *S. kraussiana*, although the shoot dichotomy regulation by auxin is questionable in this species (Sanders and Langdale, 2013).

### REGULATION OF THE APEX INTEGRITY DURING DICHOTOMY

The knowledge concerning genetic background for dichotomy regulation and molecular signaling during this process is lacking. It can only be speculated that the regulation is based on genes involved in the cell division and differentiation homeostasis, affecting the size of the apex and the identity of meristematic cells.

The genetic machinery for the self-maintenance of the apical meristems is mostly deciphered in a model plant *Arabidopsis*. Here, based on the mutant phenotypes, it is possible to infer which genes could play a role in dichotomous branching. One of such *Arabidopsis* mutants is *tonsoku* (*tsk*), with a forked root reminiscing of dichotomy, and fasciated stems. The suggested role of *tsk* is to maintain the ordered structure of the meristem through the regulation of the cell cycle (Suzuki et al., 2004, 2005). However, the disorganization in *tsk* root meristems affects the expression of the other regulatory genes, e.g., *SCARECROW* (*SCR*; Suzuki et al., 2004). Contradictory, it was shown that the tissue-specific expression pattern of the *SCR* homolog is preserved in dichotomizing roots of *Pinus*. During dichotomy progression, the specificity of endodermis and root radial patterning are maintained, manifested by localization of this gene (Laajanen et al., 2007; Raudaskoski and Salo, 2008). These results are also in agreement with the cytohistological observation of dichotomizing apices.

The maintenance of the shoot apical meristem integrity in *Arabidopsis* requires the activity of *WUSCHEL* (*WUS*), which is antagonized by the *CLAVATA* genes (Laux et al., 1996; Fletcher et al., 1999; Reddy, 2008). As these genes are involved in the production and maintenance of initial cells and their mutation can result also in bifurcated stem phenotype (e.g., in *clv3* or *WUS* overexpression mutants), their plausible role in dichotomy regulation can be therefore hypothesized. Interestingly, the homologous *CLAVATA1-LIKE* gene was found to play a potential role in the initiation of *Pinus* ectomycorrhizal roots (Heller et al., 2012). Furthermore, as recent research revealed the presence of *WUS* homologs in all plant groups (Lian et al., 2014) and the *CLAVATA3* homolog was earlier described e.g., in *Selaginella* (Floyd and Bowman, 2007), the universality of the regulatory mechanism in plants can be suggested. However, it remains unknown, whether these homologous genes have a similar expression pattern and function in the apical meristem maintenance and if they are involved in dichotomy regulation.

Recently, the WUS-CLV3 interactions were simulated in the reaction–diffusion model to show different patterns of shoot apical meristem (SAM) development (Fujita et al., 2011). The assumption was that WUS promotes the growth of the apex (activator) whereas CLV suppresses the process (inhibitor). The pattern of dichotomous branching was generated in this model, when the increased level of the activator stimulated the cell proliferation leading to the meristem bifurcation due to spatial restrictions (SAM size limitation; Fujita et al., 2011). Thus, it can imply that the proper balance of these two factors can play a role in the meristem integration during dichotomy.

Finally, the class I KNOX (*KNOTED*-like homeobox) genes are hypothesized to suppress the cell differentiation within the SAM. They are found in all land plants and, in addition, they are supposed to be regulated by hormones (Veit, 2009). For example, a low level of auxin stimulates the class I KNOX genes and in turn promotes the meristematic activity in the SAM. Likely, the formation of the new apices at the flanks of dichotomizing meristem and simultaneously triggered differentiation in its center can result from the localized distribution of auxin and related expression of genes. Furthermore, in *Selaginella KNOX/ARP* (*ASYMMETRIC LEAVES1, ROUGH SHEATH2, PHANTASTICA*) interaction regulates the maintenance of the indeterminate growth of the apex vs. leaf formation. It was suggested that this expression pattern within the SAM can be related to the meristem dichotomy (Harrison et al., 2005).

## CONCLUSION

Dichotomy is only marginally studied in plants and only its anatomical aspects are relatively well described. Nevertheless even here different definitions used and the lack of molecular background leads to the misunderstandings and erroneous interpretations.

Specification of the new ACs/meristematic centers boundaries seems to be a crucial problem for dichotomy, specifically deciphering the nature of the signal(s), the site of its origin and propagation. Likely, changes in auxin distribution and its polar transport can orchestrate the boundaries specification. The cellular and/or molecular pathways of possible auxin signaling during dichotomy, as well as its interactions with other compounds, require further research and visualization.

Determination and then separation of the meristematic centers can be related to the loss of communication between the adjacent cells of dichotomizing apices. Microsurgical and ablation experiments showed that a longitudinal split of the meristem can mimic the dichotomous branching (Snow and Snow, 1931; Reinhardt et al., 2004). However, the ablation of the superficial meristem layers did not stimulate the meristem split (Reinhardt et al., 2003) suggesting the involvement of the organizing center and the meristem identity genes expressed there.

Currently, the meristem homeostasis is extensively explored, acknowledging its vital role in plant development. The improvement of the genetic and molecular techniques, also in new model organisms, e.g., *Selaginella moellendorffii*, will enable us to fully address the problem of the meristem integrity, especially during the meristem bifurcation.

## Conflict of Interest Statement

The author declares that the research was conducted in the absence of any commercial or financial relationships that could be construed as a potential conflict of interest.
